# Pycnosomes: Condensed Endosomal Structures Secreted by *Dictyostelium* Amoebae

**DOI:** 10.1371/journal.pone.0154875

**Published:** 2016-05-17

**Authors:** Ayman Sabra, Jade Leiba, Lauriane Mas, Mathilde Louwagie, Yohann Couté, Agnès Journet, Pierre Cosson, Laurence Aubry

**Affiliations:** 1 Department of Cell Physiology and Metabolism, Faculty of Medicine, University of Geneva, 1 rue Michel Servet, CH-1211, Geneva 4, Switzerland; 2 Univ. Grenoble Alpes, F-38000 Grenoble, France; 3 CEA, BIG, Laboratoire Biologie à Grande Echelle, F-38054 Grenoble, France; 4 INSERM, U1038, F-38054 Grenoble, France; Université de Genève, SWITZERLAND

## Abstract

*Dictyostelium discoideum* has been used largely as a model organism to study the organization and function of the endocytic pathway. Here we describe dense structures present in *D*. *discoideum* endocytic compartments, which we named pycnosomes. Pycnosomes are constitutively secreted in the extracellular medium, from which they can be recovered by differential centrifugation. We identified the most abundant protein present in secreted pycnosomes, that we designated SctA. SctA defines a new family of proteins with four members in *D*. *discoideum*, and homologous proteins in other protists and eumetazoa. We developed a monoclonal antibody specific for SctA and used it to further characterize secreted and intracellular pycnosomes. Within cells, immunofluorescence as well as electron microscopy identified pycnosomes as SctA-enriched dense structures in the lumen of endocytic compartments. Pycnosomes are occasionally seen in continuity with intra-endosomal membranes, particularly in U18666A-treated cells where intraluminal budding is highly enhanced. While the exact nature, origin and cellular function of pycnosomes remain to be established, this study provides a first description of these structures as well as a characterization of reagents that can be used for further studies.

## Introduction

In eukaryotic cells, endocytosed material is rapidly delivered to membrane-delimited endocytic compartments. Endosomes are often filled with membranous material, since their limiting membrane can engage into intralumenal budding. This process can notably be stimulated by a large number of drugs, such as U18666A, although the precise mechanism of action of these drugs is poorly characterized [1]. Although they normally evolve to give rise to lysosomes, endosomes can also fuse with the plasma membrane in a regulated manner, and the membranous material released in this process is referred to as exosomes [2]. Specialized lysosomes (e.g. melanosomes of melanocytes and lytic or dense granules of hematopoietic cells) are also known to discharge their content outside the cells in response to specific external stimuli [3]. In mammalian organisms, extracellular release of exosomes or of lysosomal contents plays a major role in very diverse physiological events, such as immune response, skin pigmentation or cancer progression [3, 4].

*Dictyostelium discoideum* amoebae have been widely used to study the organization and function of the endocytic pathway, and represent a valuable model for human lysosomal and trafficking diseases [5]. In *D*. *discoideum*, internalized material (e.g. bacteria or fluids) follows the classical endosome-to-lysosome route and is then transferred to more neutral post-lysosomal compartments [6]. Approximately one hour after endocytosis, undigested remnants are released from post-lysosomes in the extracellular medium, together with some lysosomal enzymes, in a process akin to lysosome secretion in specialized mammalian cells [7]. The gene products that control the general organization and function of the endosomal and lysosomal pathways in *D*. *discoideum* and in human cells are remarkably conserved, and studies in *D*. *discoideum* cells have been instrumental in defining the specific roles of many gene products in the endocytic pathway [5]. In Dictyostelium, U18666A has been shown to induce the formation of multivesicular endosomes by stimulating intralumenal budding [8].

This study was aimed at characterizing the endocytic pathway of *D*. *discoideum* cells. We observed, by electron microcopy, the presence of dense bodies in endocytic compartments of axenically-growing *D*. *discoideum* cells, that we named pycnosomes. Pycnosomes are secreted in the extracellular medium where they accumulate and from which they can be recovered by differential centrifugation. We characterized the most abundant component of pycnosomes, the SctA protein, and produced a specific monoclonal antibody that allowed a detailed characterization of pycnosomes in *D*. *discoideum* endosomes. This report is the first description of these structures, and it provides new tools to allow future studies of pycnosomes and of the SctA protein function.

## Materials and Methods

### Cell culture and reagents

Experiments were performed on KAx-3 (from the Firtel laboratory) and DH1-10 [9] *D*. *discoideum* cells grown at 21°C in shaking suspension in HL5 axenic culture medium. When specified, U18666A (Biomol, Zürich, Switzerland) was added at a concentration of 20 μg/ml [8]. Mouse monoclonal antibodies specific for endosomal p80 (H161), and mitochondrial porin (70-100-1) were previously described [10, 11]. Hybridoma supernatants were diluted 1 in 3 before use.

### Recovery of secreted pycnosomes

*D*. *discoideum* cells were cultured in a 100 ml shaken suspension of HL5 for 4 days, reaching a cell density of 3 to 6 x 10^6^ cell/ml. A cellular pellet was recovered by centrifugation at 600 x *g* for 5 min and directly resuspended and lysed in 200 μl of denaturing reducing sample buffer. The supernatant was centrifuged at 15’000 x *g* and/or 100’000 x *g* (see figure legends) for 45 min to recover pelletable secreted material. The sedimented material was resuspended in reducing or non-reducing denaturing sample buffer for protein analysis, or in a non-denaturing buffer for further analysis, as indicated. To analyze protein composition, proteins were separated by SDS-PAGE and visualized by Coomassie blue or silver staining [12].

### Production of anti-SctA monoclonal antibody

BALB/c female mice were injected intraperitoneally with 100 μg of a pycnosomal preparation purified from *D*. *discoideum* cell culture medium and mixed with Freund's complete adjuvant. One month later, two injections (100 μg of material) in incomplete Freund's adjuvant were performed at one-week interval. Spleen cells were then extracted and fused to mouse NSI myeloma cells as described [13]. Hybridoma supernatants were assessed for the presence of anti-pycnosome antibodies using Elisa plates adsorbed with material from pycnosomes. Positive hybridoma were selected and cloned twice by limiting dilution. The SctA-specific monoclonal antibody B4.2 (IgG1) from hybridoma supernatant (respectively ascitis fluid; generated by the BIOTEM company, Apprieu, France) was used in this study without additional purification steps in immunolabeling experiments at 1/3 dilution (respectively 1/1000). The B4.2 antibody is available at the Geneva Antibody Facility (http://www.unige.ch/antibodies). All procedures regarding animal use were carried out in 1996 in the animal facility of the CEA (Grenoble, France) by qualified laboratory staff in strict accordance with the applicable European Economic Community (86–6091 EEC) guidelines for the care of laboratory animals. A standard immunization protocol was followed, inoculating three 12-week old mice. Animals had free access to food and water and were subjected to daily surveillance to detect any sign of animal suffering (weight loss; exacerbated inflammatory reaction at injection site; prostration; absence of self-grooming; abnormal behavior) that would have led to animal euthanasia before the end of the protocol. Animals were sacrificed by CO2 inhalation.

### Plasmid constructs and recombinant protein purification

The cDNA of SctA (DDB_G0278725, Genbank accession number O77257) and SctB (DDB_G0291255) truncated of the 57 first base pairs (corresponding to the predicted signal peptide) were subcloned in the pGEX-KG plasmid in frame with the N-terminal GST. The constructs that required PCR amplification steps were verified by sequencing. The recombinant proteins (GST-SctA and GST-SctB) were expressed in *E*. *coli* Bl21(DE3) bacteria at 37°C (0.1 mM IPTG, 3h) and purified by affinity on a gluthatione sepharose column according to the manufacturer’s instructions (GE healthcare, Orsay, France).

### Immunofluorescence microscopy

Immunofluorescence analysis was performed essentially as described [14]. Briefly, *D*. *discoideum* cells were allowed to attach to a glass coverslip for 30 min at room temperature and then fixed 10 min with 4% paraformaldehyde. Fixed cells were washed twice in Phosphate Buffered Saline (PBS), permeabilized in methanol at -20°C for 2 minutes, and then washed twice in PBS and once in PBS containing 0.2% (w/v) bovine serum albumin (PBS-BSA). Permeabilized cells were incubated with the anti-SctA antibody (B4.2) for 1h, washed twice in PBS-BSA, and then stained with an Alexa488 fluorescent secondary antibody. Labeled cells were then incubated with an anti-p80 monoclonal antibody (H161) directly coupled to Alexa fluor 647. Cells were observed on a laser-scanning confocal microscope (Zeiss LSM 700).

### Electron microscopy

Cellular or secreted material pellets were fixed for 30 min in HL5 medium containing 2% glutaraldehyde and then incubated further in 2% glutaraldehyde-containing phosphate buffer (0.1 M, pH 7.4) for 1 h. After three washes in phosphate buffer, pellets were either treated with 0.3% of cold osmium and embedded in Epon resin for conventional electron microscopy [15], or processed for cryosectioning as described previously [16]. Frozen sections were transferred to grids and incubated with either the H161 monoclonal antibody specific for the p80 endosomal protein or with the B4.2 antibody. Then, secondary gold-coupled antibodies to mouse immunoglobulins were used.

## Results

### Dense bodies in *D*. *discoideum* endocytic compartments

**Fig 1:**
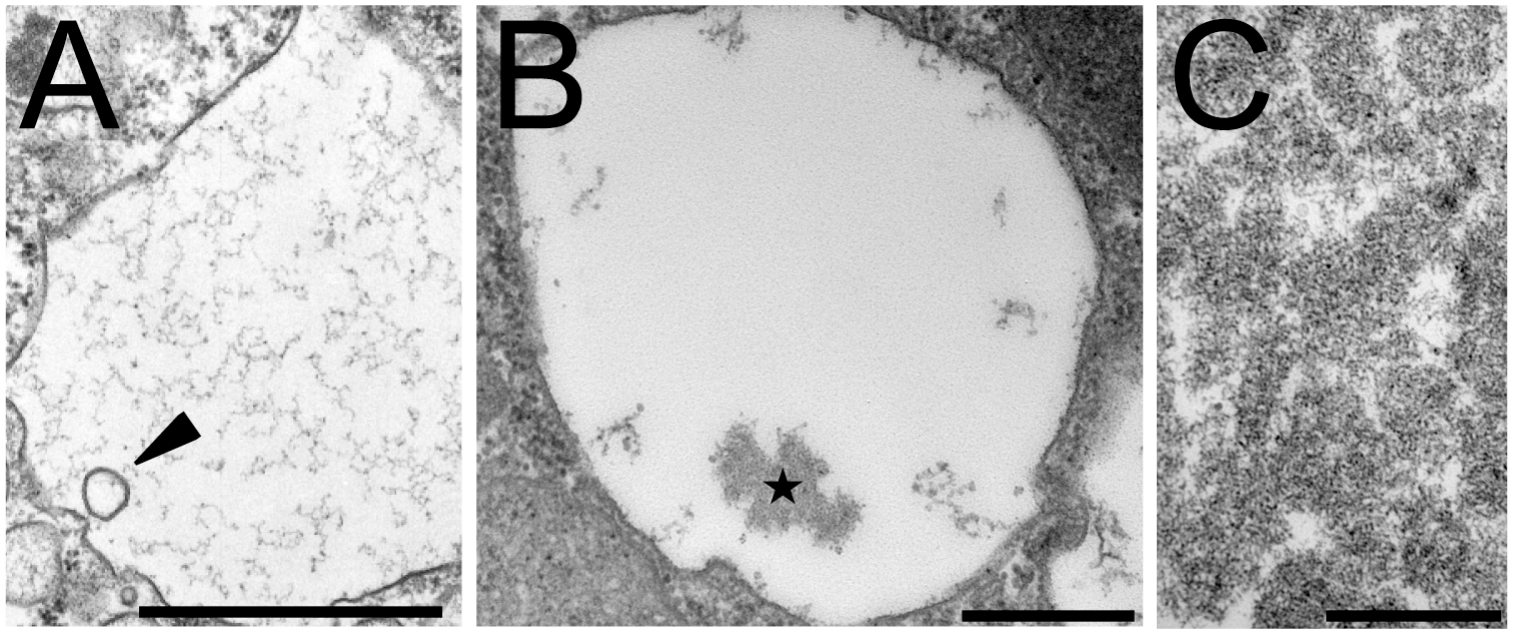
Pycnosomes present in *D*. *discoideum* endocytic compartments are secreted in the extracellular medium. *D*. *discoideum* cells grown in axenic medium were fixed and processed for electron microscopy. (A) Most endocytic compartments appeared empty or contained occasionally a few vesicles (arrowhead). (B) Dense bodies (pycnosomes) appeared as amorphous structures in the endosomal lumen (star). (C) Secreted pycnosomes were recovered from the extracellular medium by differential centrifugation. Bars: 500 nm.

In *D*. *discoideum* cells grown in liquid HL5 medium, endocytic compartments observed by electron microscopy appear essentially as large, mostly empty compartments [8]. Exceptionally, a few intraluminal vesicles can be observed (Fig 1A, arrowhead). A more detailed examination also revealed the frequent presence of mostly amorphous electron-dense structures (Fig 1B, star). These structures were named pycnosomes (from the greek pycnos, dense). To further characterize pycnosomes, we reasoned that they may be secreted in the extracellular medium since *D*. *discoideum* endocytic compartments continuously fuse with the plasma membrane. Cells were therefore pelleted at low speed (600 x *g*) and the material present in the cell supernatant was recovered by centrifugation at higher speed (15’000 x *g* or 100’000 x *g*). When fixed and processed for electron microscopy, the 15’000 x *g* pellet appeared very homogeneous with almost exclusively dense structures very similar to those observed in cells (Fig 1C). This observation indicates that pycnosomes are secreted in the extracellular medium and that a highly enriched preparation of pycnosomes can be obtained by centrifugation of the cell culture medium.

### SctA is the most abundant protein in secreted pycnosomes

**Fig 2:**
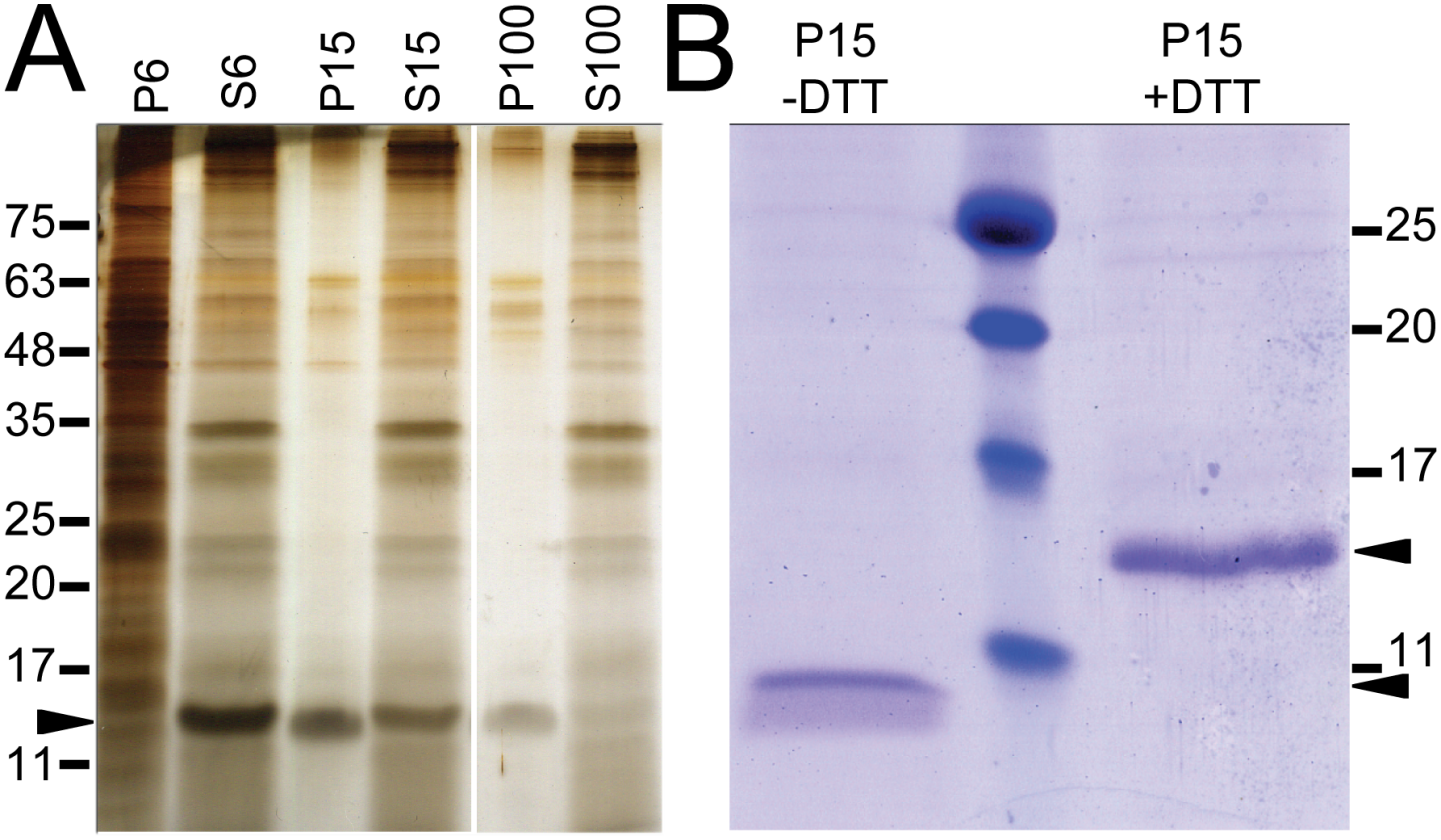
SctA is a major constituent of secreted dense bodies. (A) *D*. *discoideum* cells were pelleted by centrifugation at 600 x g (pellet P6) and the supernatant (S6) was sequentially subjected to centrifugations at 15’000 and 100’000 x g. The corresponding supernatants (S15, S100) and pellets (P15, P100) resuspended in an equivalent volume were diluted in reducing sample buffer and separated on a 15% acrylamide gel. Equal volume of samples were loaded except for P6 that was diluted 1/10. The main proteins were revealed by silver staining. (B) The same amount of the 15 000 x g pellet was resuspended in reducing (+DTT) or non reducing (-DTT) sample buffer and analyzed by SDS-PAGE and Coomassie staining. Molecular weights (in kDa) are indicated, as well as the SctA protein (arrowheads).

To characterize the protein composition of pycnosomes, they were purified as described above and were analyzed by SDS-PAGE electrophoresis and silver staining. One main protein was detected in the 15’000 x *g* pellet at an approximate molecular weight (MW) of 15 kDa, and it was almost fully sedimented after the 100’000 x *g* centrifugation (Fig 2A, arrowhead). This protein was named SctA, an abbreviation for Secreted Protein A. The identity of SctA was determined by mass spectrometry (MS) analysis of the corresponding band extract (S1 Table) and matched the *D*. *discoideum* gene product previously named p17 (DDB_G0278725)[17].

**Fig 3:**
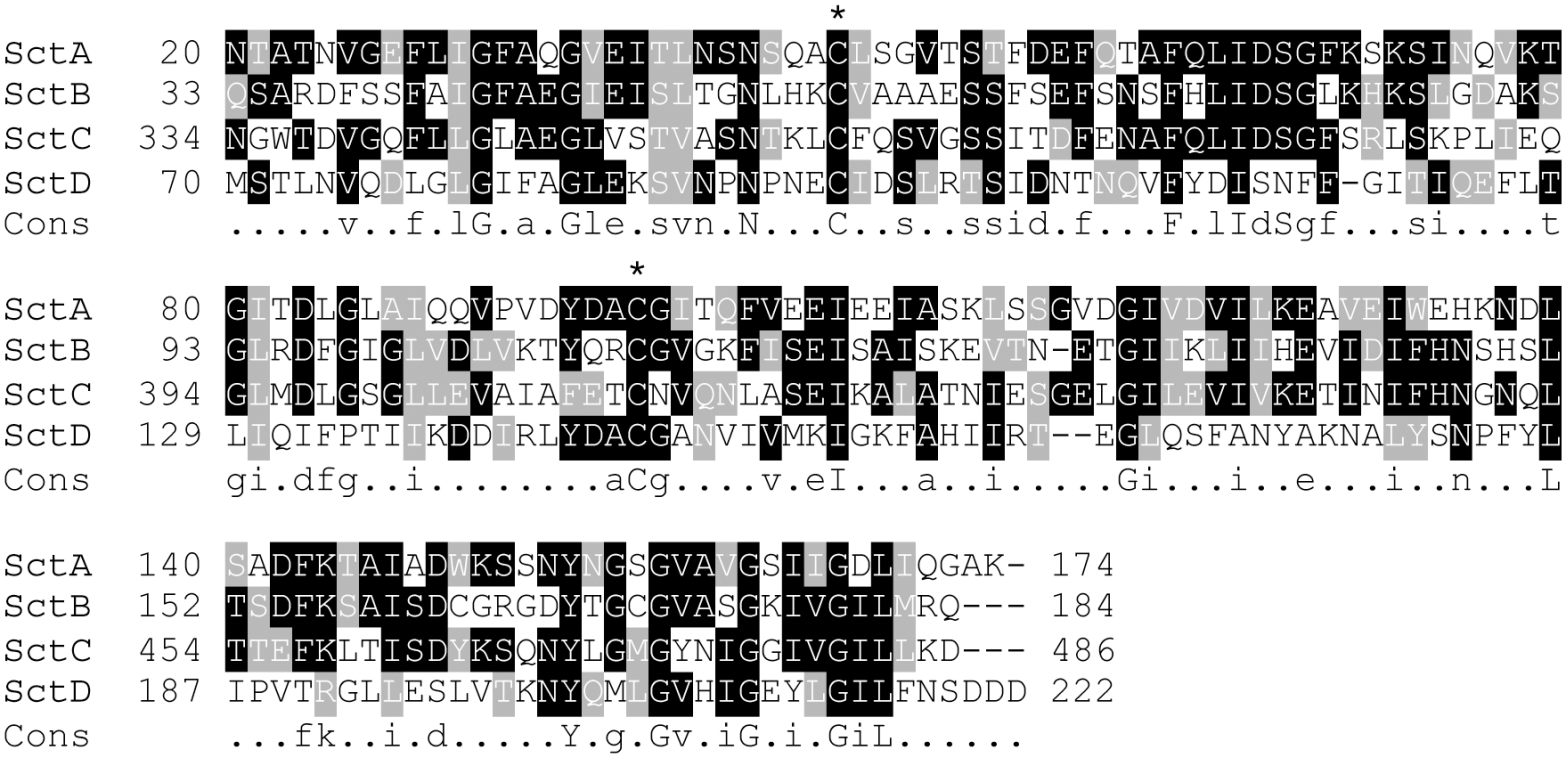
The Sct family of proteins. The SctA protein was identified by mass spectrometry as the product of the DDB_G0278725 gene. The *D*. *discoideum* genome encodes three proteins exhibiting sequence homology to SctA: SctB, SctC and SctD. All Sct proteins harbor a signal peptide (not shown) and a conserved pair of cysteines residues (*). The regions of homology between all Sct proteins were aligned using the Multalin software and treated with Boxshade. The consensus sequence is indicated. The SctC protein contains a long extension rich in glycines and serines, just downstream of the predicted signal peptide (not shown on the figure).

The *sctA* gene consists in a single exon of 525 bp and encodes a 174 amino-acid protein (Fig 3). Analysis of the protein sequence using SignalP [18] predicted an N-terminal signal peptide cleaved at the carboxyl side of Ala_19_. This cleavage was confirmed by sequencing of the N-terminal peptide that starts at Asn_20_ (data not shown). The estimated molecular weight of the predicted mature protein is 16.4 kDa. The SctA protein contains no predicted transmembrane domains or any conserved functional or structural domain. The presence of two Cys residues at positions 47 and 98 prompted us to test their participation in the tertiary structure of SctA. Under non-reducing conditions, the apparent molecular mass of SctA was noticeably modified with a shift from approximately 15 kDa to 10 kDa (Fig 2B). This difference in the electrophoretic mobility strongly suggests the presence of an intramolecular disulfide bond.

We searched protein databases with psi-BLAST and identified after six iterations a stable list of 75 proteins with a significant degree of similarity with SctA (S2 Table) in *D*. *discoideum* and in other species, ranging from protists to eumetazoa. No functional or structural information is available to date on any member of this family. In *D*. *discoideum*, three predicted proteins present a significant homology to SctA and are referred to here as SctB (DDB_G0291255, previously called 29C), SctC (DDB_G0287399), and SctD (DDB_G0284199) (Fig 3). Like SctA, SctB, C and D harbor a predicted signal peptide expected to be cleaved, leading to theoretical MW for the mature forms of about 18, 44 and 23 kDa respectively. The last C-terminal 150 residues of SctB, C and D share 39%, 39% and 23% identity with SctA respectively, including the two Cys residues mentioned above (Fig 3). In SctC a domain enriched in Serine and Glycine residues is present at the N-terminus of the mature protein and accounts for the higher predicted MW of the protein. Analysis by mass spectrometry of the 600 x *g* cell supernatant identified the presence of SctB and C proteins in addition to SctA, indicating that these two proteins at least are expressed in axenic growth conditions and secreted in the extracellular medium (S1 Table). Available RNA Sequence data suggests that SctA, B and C are abundantly expressed in growing cells, and repressed during multicellular development [19]. The propensity of SctB and SctC to associate with secreted pycnosomes remains to be determined.

Proteins presenting a significant degree of similarity were also identified in other Dictyostelids (*Acytostelium subglobosum*, *Polysphondylium pallidum*, *Dictyostelium fasciculatum*, *Dictyostelium purpureum*), in ciliates (*Oxytricha trifallax*, *Stylonychia lemnae*, *Paramecium tetraurelia*), in other protozoa (*Naegleria gruberi*, *Thecamonas trahens*, *Vitrella brassicaformis*) as well as in eumetazoa (*Strongylocentrotus purpuratus*, *Nematostella vectensis*, *Lingula anatina*, *Hydra vulgaris*, *Branchiostoma floridae*) (S2 Table, S2 Fig). This suggests that the Sct proteins form a conserved family of proteins, probably sharing a common structure, extending from protists to eumetazoa.

### Immunodetection of endogenous SctA in *D*. *discoideum* pycnosomes

**Fig 4:**
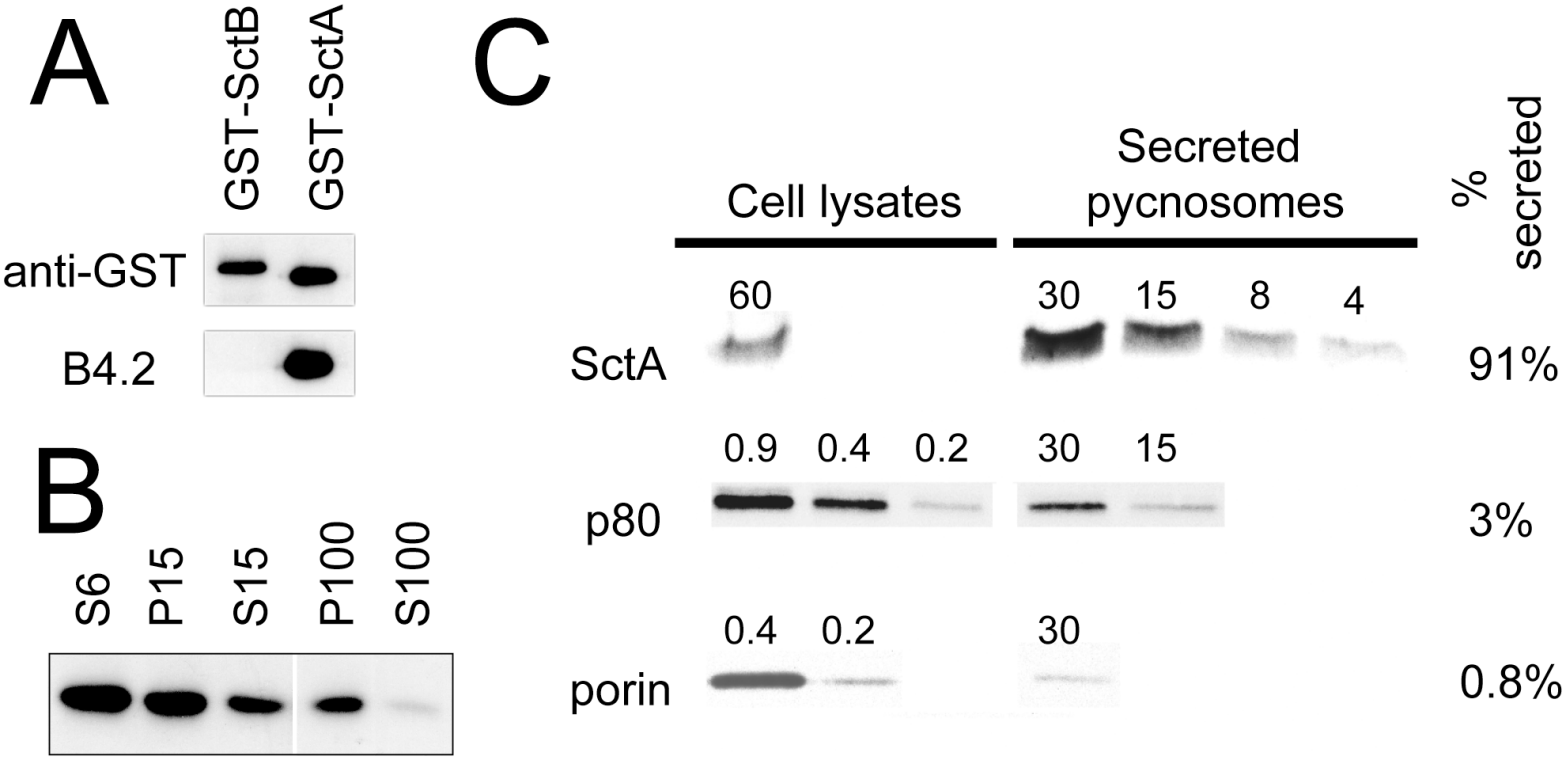
SctA is massively associated with secreted pycnosomes. (A) The SctA and B proteins were expressed in bacteria as GST-fusion proteins, purified, and analyzed by Western blot with anti-GST and B4.2 antibodies. While an anti-GST antibody labeled both proteins, the B4.2 antibody only recognized GST-SctA and not SctB. (B) Supernatants (S6, S15, S100) and corresponding pellets (P15, P100) obtained by differential centrifugation of *D*. *discoideum* cell culture medium (600, 15’000, 100’000 x *g*, see Fig 2) were analyzed by Western blot using the B4.2 antibody. Equal volumes were loaded for all samples. (C) *D*. *discoideum* cells were cultured for 4 days. Cells and a fraction enriched in secreted pycnosomes were recovered by successive centrifugation of cell suspension at 600 x *g* and 100’000 x *g* respectively. Serial two-fold dilutions from the two fractions were analyzed by Western blot using the B4.2 antibody, the H161 anti-p80 and an antibody against mitochondrial porin. The number of cells loaded on each lane is indicated above each band. The fraction of each protein associated to secreted pycnosomes is indicated on the right.

A pycnosome-enriched preparation was used to immunize mice and to produce monoclonal antibodies, leading to the selection of the clone B4.2. The specificity of the B4.2 towards SctA was assessed using a recombinant form of the protein fused to the glutathione S-transferase. As a control, a GST-fusion of SctB was used in parallel. The B4.2 antibody specifically recognized purified GST-SctA, but not GST-SctB (Fig 4A). By Western blot analysis, B4.2 identified the SctA protein at the expected MW in the extracellular material and in purified pycnosomes (Fig 4B).

The relative abundance of intracellular *versus* secreted SctA was determined by comparing SctA distribution in serial two-fold dilutions of a cell lysate and of a 100’000 x *g* pellet obtained from a cell supernatant and that contains most of the secreted SctA protein. After four days of culture, approximately 90% of the total SctA was found in the 100’000 x *g* pellet, and 10% in the cellular lysate. Only traces of the endosomal p80 protein (3%) and of a mitochondrial porin (0.8%) were detected in the 100’000 x *g* fraction. (Fig 4C). These results indicate that SctA is massively secreted in the extracellular medium in a pelletable fraction.

**Fig 5:**
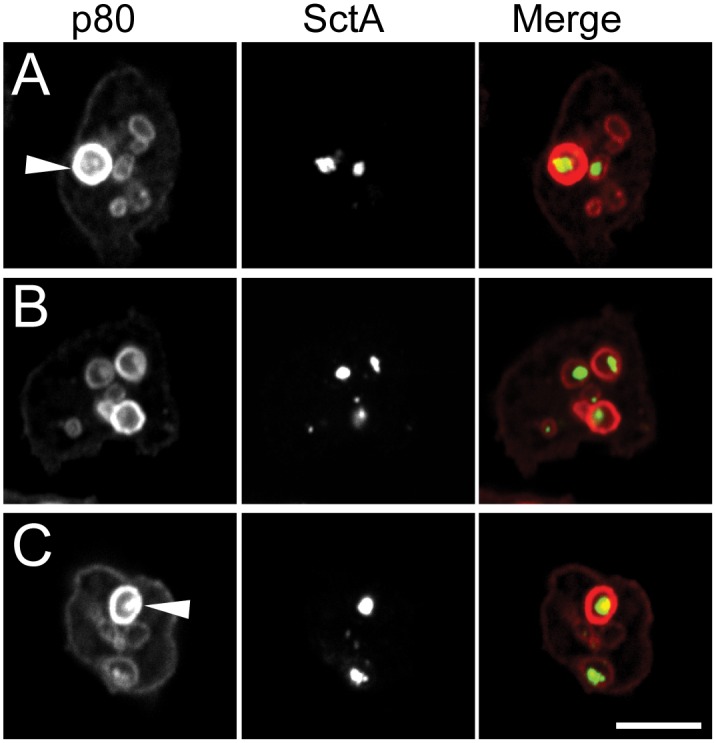
SctA-positive structures are detected in endocytic compartments. Confocal images of three *D*. *discoideum* cells, labeled for SctA protein (middle column, green) and for endosomal p80 (left column, red) and merged images (right column) are shown. SctA-positive structures were detected both in large p80-rich post-lysosomes (arrowheads) and in p80-low endosomes. Bar: 10 μm.

To assess the subcellular localization of SctA, *D*. *discoideum* cells were fixed, permeabilized and stained with the B4.2 antibody. SctA was detected in numerous discrete structures (Fig 5). Co-staining with the antibody H161 directed against p80, that is present in all endocytic compartments [11], indicated that SctA-enriched structures are localized within compartments of the endocytic pathway (Fig 5). More specifically, SctA was detected both in large p80-rich post-lysosomes and in p80-low endocytic compartments. In agreement with this observation, SctA was found to co-distribute with endocytic compartments after separation of cellular compartments on a Percoll gradient (S1 Fig).

**Fig 6:**
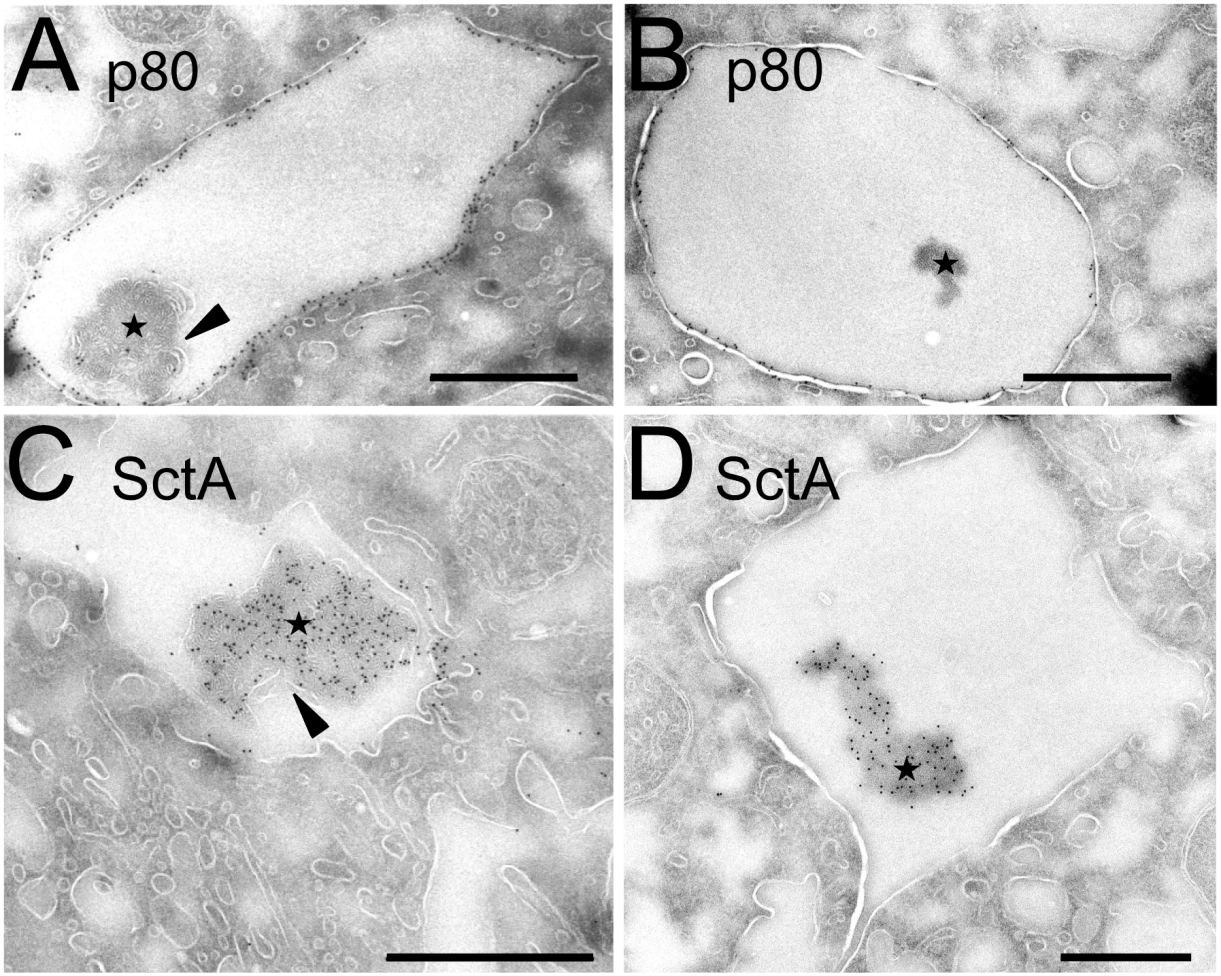
Electron microscopy reveals SctA-enriched endosomal pycnosomes. *D*. *discoideum* cells grown in axenic medium were processed for immuno-electron microscopy. (A-B) Sections were labeled with the H161 anti-p80 antibody. The p80 protein was abundantly present in endosomal membranes, and only small amounts of p80 were found associated with pycnosomes (stars) in the lumen. (C-D) The B4.2 antibody revealed a high concentration of SctA in endosomal pycnosomes. In some pictures, pycnosomes appeared associated with some membranous elements (arrowheads). Bar: 500 nm.

To observe with a better resolution the localisation of SctA inside endosomal compartments, *D*. *discoideum* cells were fixed and processed for cryosectioning and immuno-electron microscopy. The B4.2 and the H161 antibodies were used to label SctA and p80, respectively. In cryosections, dense structures similar to pycnosomes described above in Epon-embedded samples were seen inside endosomal compartments positive for p80. These structures were essentially devoid of p80 labeling (Fig 6A and 6B, stars), but were highly enriched in SctA (Fig 6C and 6D, stars). Together, our results establish that SctA is a specific marker of pycnosomes present in endocytic compartments and secreted in the extracellular medium.

### Association of pycnosomes with intra-endosomal membranes

**Fig 7:**
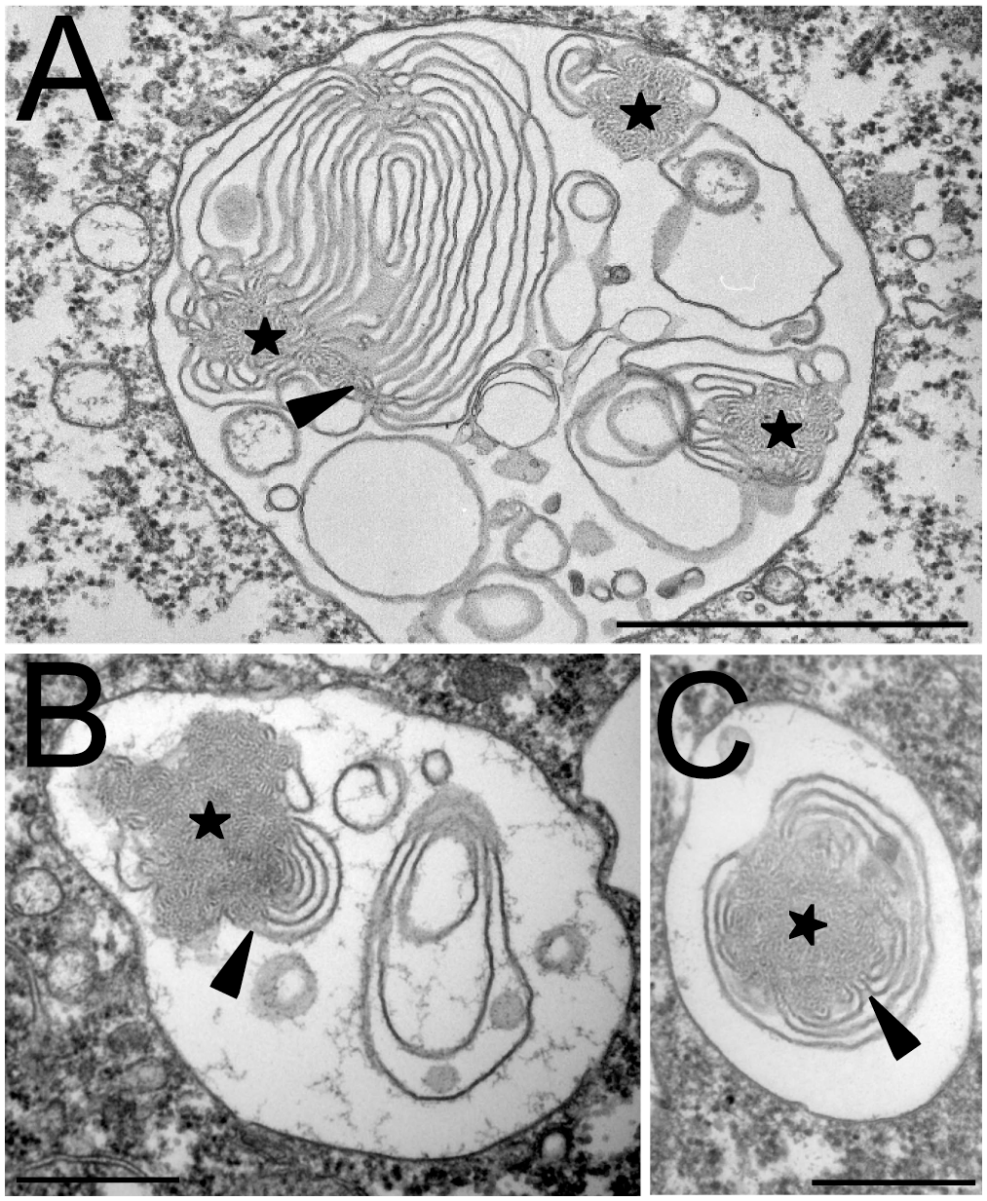
Intraendosomal membranes association with pycnosomes. *D*. *discoideum* cells were treated for 2h with U18666A to induce the formation of intraluminal membranes in the endocytic compartments, then fixed and processed for electron microscopy. Pycnosomes (stars) often appeared continuous with internal membranes. Arrowheads point to regions where the continuity between pycnosomal material and endosomal membranes was most apparent. Bar: 500 nm.

**Fig 8:**
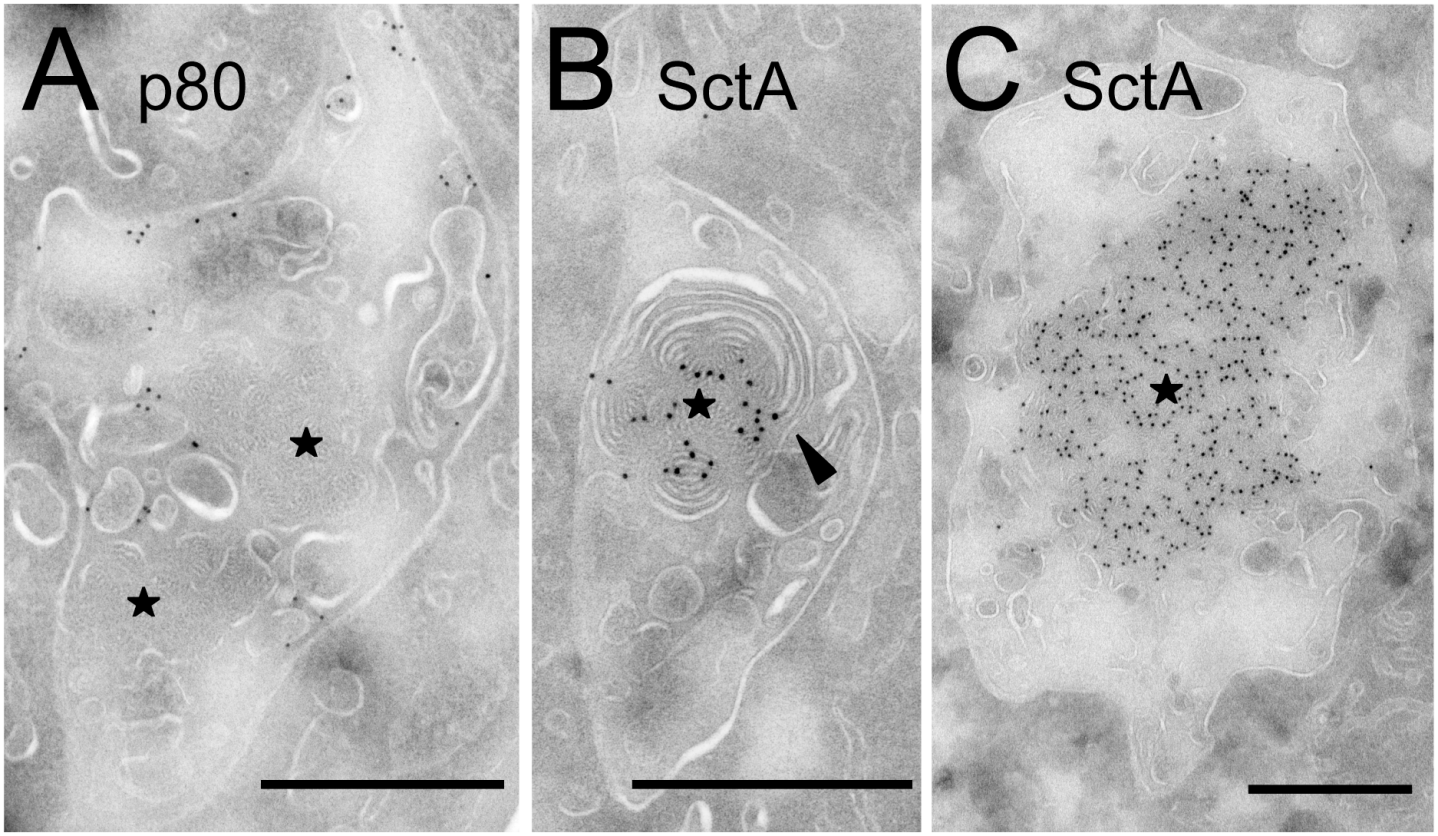
SctA-positive material appears continuous with intraendosomal membranes. *D*. *discoideum* cells were treated for 2h with U18666A, then fixed and processed for immuno-electron microscopy. (A) In sections labeled with the H161 anti-p80 antibody, the p80 protein was seen associated with the limiting endosomal membrane as well as with internal membranes, but was not detected in pycnosomes (stars). (B-C) SctA-positive structures with the dense morphology of pycnosomes were also detected, and often appeared continuous with intraluminal membranes (arrowhead). Bar: 500 nm.

While pycnosomes appeared mostly composed of amorphous material, in a few exceptional instances they appeared to be continuous with membranous material at their periphery (arrowheads in Fig 6A and 6C). In order to investigate the putative relationship between pycnosomes and intraendosomal membranes, we treated *D*. *discoideum* cells for 2h with U18666A to stimulate intraendosomal budding [8]. In these conditions, pycnosomes were frequently seen associated with intraendosomal membranes in Epon-embedded cells (Fig 7) and some pictures suggested a direct continuity between pycnosomal material and surrounding membranes (arrowheads). Staining of thin sections with antibodies confirmed that in these conditions, pycnosomes were still devoid of p80 (Fig 8A) and enriched in SctA (Fig 8B and 8C). As discussed below, the apparent continuity between SctA-enriched dense bodies and intraendosomal membranes may suggest that pycnosomes arise by condensation of membranes within *D*. *discoideum* endocytic compartments, although this remains to be firmly established.

## Discussion

We report here the first observation of a novel structure in the lumen of *D*. *discoideum* endocytic compartments that we named *pycnosomes*. Pycnosomes are massively secreted in the extracellular medium from which they can be recovered by differential centrifugation. We identified the protein SctA as a marker of both intracellular and secreted pycnosomes. In cells, SctA is abundantly and virtually exclusively found associated with intra-endosomal pycnosomes. This protein belongs to a novel family of proteins present in protists but also in some metazoans.

Based on the observations presented, two different scenarios can be proposed regarding the formation and the nature of pycnosomes. According to the first scenario, pycnosomes may arise by the accretion and condensation of internal endocytic membranes. This is supported by the fact that pycnosomes sometimes appear to be continuous with intra-endosomal membranes, notably in cells treated with U18666A in which intraluminal membranes are particularly abundant. In untreated cells association of pycnosomes with membranes was not frequently seen, and this may reflect the fact that they represent a transient state in the formation of mature pycnosomes. In this model, SctA could either adsorb non specifically to the membrane remnants or contribute to the pycnosome formation as an integral constituent and therefore gradually concentrate as membranous material condenses. An alternative scenario proposes that pycnosomes could arise by direct aggregation of SctA (and possibly a few other lumenal components), possibly due to the specific pH and ionic composition of endocytic compartments. This would account for the observation that SctA is extremely concentrated in pycnosomes. Proteinaceous condensed structures have been observed in various organelles, for example in mammalian cells in regulated secretory vesicles containing insulin. According to this second model, such protein aggregates would only accidentally come into direct contact with neighboring intraluminal membranes. Further biochemical analysis, such as determination of the lipid composition, may shed new light on the structure and origin of pycnosomes.

At this stage, we can only speculate about the role of pycnosomes, based on the observation that they are secreted by cells into the extracellular medium. They may represent cellular waste products. Alternatively, secreted pycnosomes may come into contact with other cells, and thus allow a form of intercellular communication. Secreted pycnosomes may also play a role in cellular adhesion by forming a matrix on extracellular surfaces. In order to better understand the nature and function of pycnosomes, and the role of the SctA protein, we attempted to delete the SctA gene by homologous recombination. So far, these attempts have been unsuccessful, maybe indicating that the *sctA* gene is essential for cell survival.

The availability of a specific monoclonal antibody against SctA provides a valuable tool to further investigate the role of SctA and of pycnosomes. In particular, it will allow an easy characterization of pycnosomes in specific mutants of the *D*. *discoideum* amoeba. It will also be interesting to determine how pycnosomes and SctA behave in cells grown in the presence of bacteria, where extensive multilamellar endosomes are observed [8]. In the future, this may reveal unsuspected mechanisms governing the formation and function of pycnosomes and of its main protein constituent, and unveil new mechanisms at play in the endocytic pathway of *D*. *discoideum*.

## Supporting Information

S1 Fig SctA co-distributes with endocytic compartments on a Percoll gradient. A post-nuclear supernatant of FITC-dextran loaded *D*. *discoideum* cells was fractionated on a 24% Percoll gradient. After elution, fractions were analyzed by spectrofluorimetry and Western blot to quantify the endocytic marker FITC-dextran and SctA respectively.
(TIF)

S2 Fig Conservation of SctA structural domain. The most divergent members of the SctA family (from dictyostelids, ciliates, hydrozoans, sea anemones and sea urchins) were aligned to identify the most conserved residues in this domain. Only the conserved regions is shown. The detailed identity of the aligned proteins can be found in S2 Table.
(TIF)

S1 Table Sct Peptides identified by mass spectrometry. (DOCX)

S2 Table A complete list of proteins exhibiting homology to SctA. (DOCX)
