# Supplementary figures and images for: Pycnosomes: Condensed Endosomal Structures Secreted by Dictyostelium Amoebae
Source: PLoS One. 2016 May 17;11(5):e0154875. doi: 10.1371/journal.pone.0154875 (PMC4871501; doi:10.1371/journal.pone.0154875)

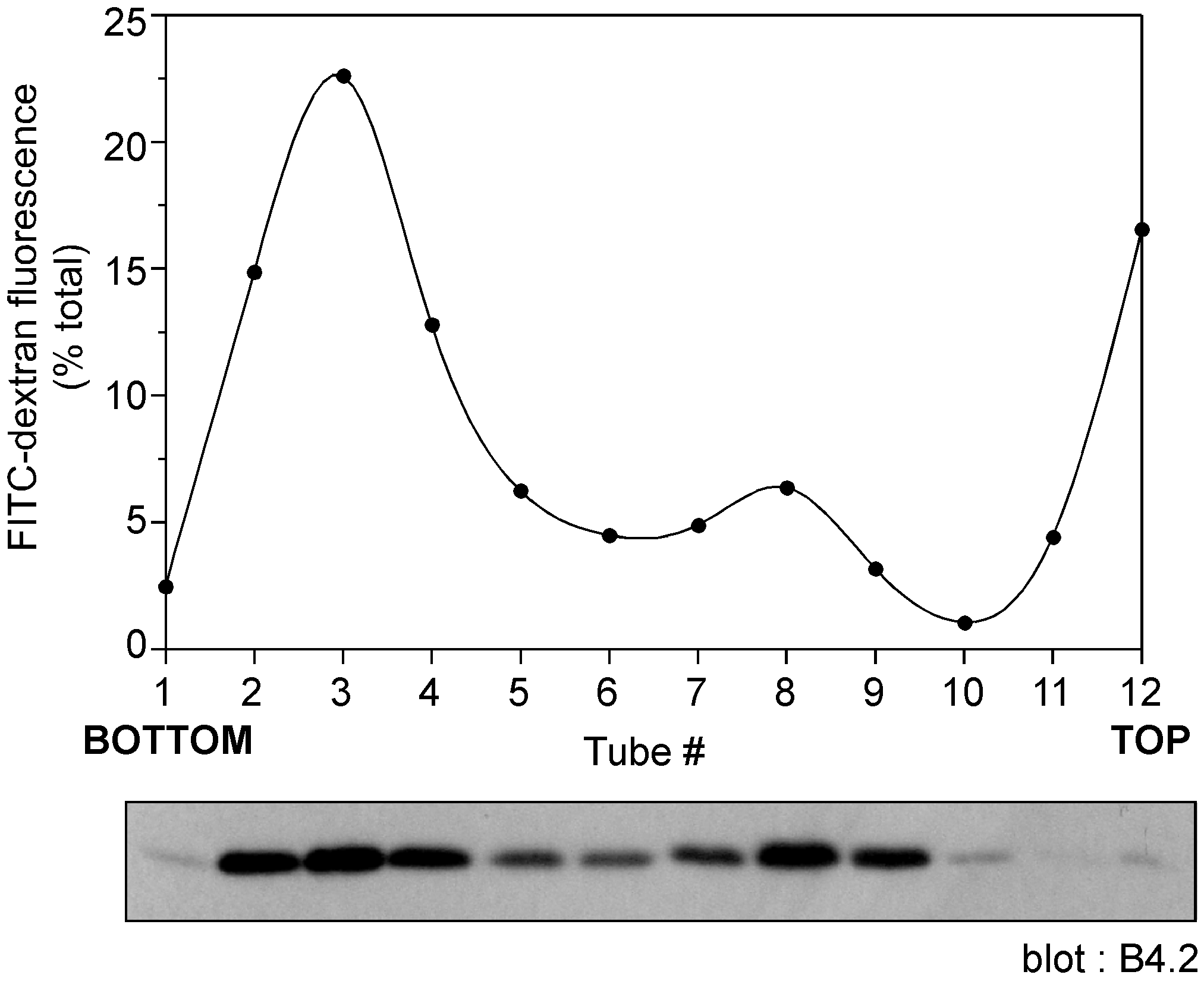

Supplement: S1 Fig — A post-nuclear supernatant of FITC-dextran loaded D. discoideum cells was fractionated on a 24% Percoll gradient. After elution, fractions were analyzed by spectrofluorimetry and Western blot to quantify the endocytic marker FITC-dextran and SctA respectively. (TIF) [file pone.0154875.s001.tif]
