# Supplementary material for: Pycnosomes: Condensed Endosomal Structures Secreted by Dictyostelium Amoebae
Source: PLoS One. 2016 May 17;11(5):e0154875. doi: 10.1371/journal.pone.0154875 (PMC4871501; doi:10.1371/journal.pone.0154875)
Supplement: S1 Table — (DOCX) [file pone.0154875.s003.docx]

**Supporting Table 1**

**S1 Table:**

**Peptides identified by mass spectrometry**

**SctA:** EAVEIWEHK

EAVEIWEHKNDLSADFK*

LSSGVDGIVDVILK

NDLSADFK

NDLSADFKTAIADWK*

SSNYNGSGVAVGSIIGDLIQGAK

TAIADWK

TAIADWKSSNYNGSGVAVGSIIGDLIQGAK*

TGITDIGIAIQQVPVDYDACGITQFVEEIEEIASK

**SctB:** DFGIGLVDLVK

EVTNETGIIK

FISEISAISK

GDYTGCGVASGK

IVGILMR

IVGILMRQ*

NEVSQTELQSAR

**SctC:** ETINIFHNGNQLTTEFK

* missed cleavage of tryptic peptide

**Materials and Methods:**

SDS-PAGE separated proteins (4-12% NuPAGE gel, Invitrogen) were stained with R-250 Coomassie blue (BioRad) before in-gel digestion using modified trypsin (Promega, sequencing grade), as described in Casabona MG et al. (Proteomics, 2013). Resulting peptides were analysed by online nanoLC-MS/MS (UltiMate 3000 coupled to LTQ-Orbitrap Velos pro, Thermo Scientific). Peptides and proteins were identified and quantified using the MaxQuant software (version 1.5.2.8) (Cox and Mann, Nat. Biotechnol. 2008) through concomitant searches against dictyBase, Uniprot (*Bos taurus* and *Saccharomyces cerevisiae* s288c taxonomies) (August 2015 versions) and the frequently observed contaminant database embedded in MaxQuant. Minimum peptide length was set to 7 amino acids. Minimum number of peptides, razor + unique peptides and unique peptides were all set to 1. Maximum false discovery rates were set to 0.01 at peptide and protein levels.
