# Supplementary material for: Pycnosomes: Condensed Endosomal Structures Secreted by Dictyostelium Amoebae
Source: PLoS One. 2016 May 17;11(5):e0154875. doi: 10.1371/journal.pone.0154875 (PMC4871501; doi:10.1371/journal.pone.0154875)
Supplement: S2 Table — (DOCX) [file pone.0154875.s004.docx]

**Supporting Table 2**

**S2 Table: A complete list of proteins exhibiting homology to SctA.**

DICTYOSTELIDS

Acytostelium subglobosum (cellular slime molds)

ref|XP_012759838.1|

ref|XP_012755351.1|

Dictyostelium discoideum (cellular slime molds)

ref|XP_647791.1| SctA

ref|XP_637288.1| SctC

ref|XP_635104.1| SctB

ref|XP_638639.1| SctD

Polysphondylium pallidum (cellular slime molds)

gb|EFA76994.1|

gb|EFA76764.1|

gb|EFA77070.1|

gb|EFA77407.1|

gb|EFA76995.1|

Dictyostelium fasciculatum (cellular slime molds)

ref|XP_004352599.1|

ref|XP_004366185.1|

ref|XP_004355485.1|

ref|XP_004366752.1|

ref|XP_004360753.1|

ref|XP_004356070.1|

ref|XP_004366751.1|

ref|XP_004354894.1|

ref|XP_004359243.1|

ref|XP_004350037.1|

Dictyostelium purpureum (cellular slime molds)

ref|XP_003287877.1|

ref|XP_003289267.1|

ref|XP_003292553.1|

ref|XP_003288542.1|

ref|XP_003287665.1|

ref|XP_003283712.1|

CILIATES

Oxytricha trifallax (ciliates)

gb|EJY72073.1|

gb|EJY72245.1|

gb|EJY73217.1|

gb|EJY85710.1|

gb|EJY74774.1|

gb|EJY88078.1|

gb|EJY67292.1|

gb|EJY81370.1|

gb|EJY77469.1|

gb|EJY74775.1|

gb|EJY77719.1|

gb|EJY83605.1|

gb|EJY72613.1|

gb|EJY76899.1|

gb|EJY81694.1|

gb|EJY70535.1|

gb|EJY83844.1|

gb|EJY81992.1|

gb|EJY77132.1|

Stylonychia lemnae (ciliates)

emb|CDW82544.1|

emb|CDW72936.1|

emb|CDW78581.1|

emb|CDW75594.1|

emb|CDW71791.1|

emb|CDW82039.1|

emb|CDW90776.1|

emb|CDW85994.1|

emb|CDW82271.1|

emb|CDW90839.1|

emb|CDW81155.1|

emb|CDW82269.1|

emb|CDW91367.1|

Paramecium tetraurelia (ciliates)

ref|XP_001426301.1|

ref|XP_001459510.1|

ref|XP_001459520.1|

OTHER PROTOZOA

Naegleria gruberi (eukaryotes)

ref|XP_002679021.1|

Thecamonas trahens (eukaryotes)

gb|KNC48820.1|

Vitrella brassicaformis (eukaryotes)

emb|CEM35767.1|

EUMETAZOA

Strongylocentrotus purpuratus (sea urchins)

ref|XP_003725880.2|

ref|XP_011673838.1|

ref|XP_003723969.2|

ref|XP_011675557.1|

ref|XP_011674213.1|

ref|XP_800047.3|

Nematostella vectensis (sea anemones)

ref|XP_001628477.1|

Lingula anatina (brachiopods)

ref|XP_013392878.1|

ref|XP_013392877.1|

Hydra vulgaris (hydrozoans)

ref|XP_004207957.1|

Branchiostoma floridae (lancelets)

ref|XP_002592327.1|

ref|XP_002609003.1|
